# Revisiting the Drasdo Model: Implications for Structure-Function Analysis of the Macular Region

**DOI:** 10.1167/tvst.9.10.15

**Published:** 2020-09-14

**Authors:** Giovanni Montesano, Giovanni Ometto, Ruth E. Hogg, Luca M. Rossetti, David F. Garway-Heath, David P. Crabb

**Affiliations:** 1 City, University of London–Optometry and Visual Sciences, London, UK; 2 NIHR Biomedical Research Centre, Moorfields Eye Hospital NHS Foundation Trust and UCL Institute of Ophthalmology, London, UK; 3 Centre for Public Health, Queen's University Belfast, Block B, Royal Hospital, Grosvenor Road, Belfast, Northern Ireland; 4 University of Milan–ASST Santi Paolo e Carlo, Milan, Italy

**Keywords:** Drasdo model, glaucoma, structure-function, macula, retinal ganglion cells

## Abstract

**Purpose:**

To provide a consistent implementation of a retinal ganglion cell (RGC) displacement model proposed by Drasdo et al. for macular structure-function analysis, customizable by axial length (AL).

**Methods:**

The effect of axial length on the shape of the inner retina was measured on 235 optical coherence tomography (OCT) scans from healthy eyes, to provide evidence for geometric scaling of structures with eye size. Following this assumption, we applied the Drasdo model to map perimetric stimuli on the radially displaced RGCs using two different methods: Method 1 only displaced the center of the stimuli; Method 2 applied the displacement to every point on the edge of the stimuli. We compared the accuracy of the two methods by calculating, for each stimulus, the number of expected RGC receptive fields and the number RGCs calculated from the histology map, expected to be equivalent. The same calculation was repeated on RGC density maps derived from 28 OCT scans from 28 young healthy subjects (age < 40 years) to confirm our results on clinically available measurements.

**Results:**

The size of the retinal structures significantly increased with AL (*P* < 0.001) and was well predicted by geometric scaling. Method 1 systematically underestimated the RGC counts by as much as 60%. No bias was observed with Method 2.

**Conclusions:**

The Drasdo model can effectively account for AL assuming geometric scaling. Method 2 should be used for structure-function analyses.

**Translational Relevance:**

We developed a free web App in Shiny R to make our results available for researchers.

## Introduction

The health of the macula is of central importance for everyday functions, such as reading and recognizing faces.^1^^–^^3^ It is now recognized that the macula can be affected by glaucoma, even in the early stages of the disease process.^4^ Loss and dysfunction of retinal ganglion cells (RGCs) in glaucoma is monitored using both structural and functional measurements. Structural assessment of the macular region can be done by spectral domain optical coherence tomography (SD-OCT), which provides volumetric measurements of various retinal layers. The layers of most interest for glaucoma are the retinal nerve fiber layer (RNFL), the ganglion cell layer (GCL), and the inner plexiform layer (IPL). Together, they form the inner retina. RGC loss from glaucoma causes thinning of the RNFL (which contains RGC axons), the GCL (which contains RGC cell bodies), and the IPL (which contains the RGC dendritic arbors).

Functional assessment for glaucoma is typically assessed with the visual field (VF) test in the form of standard automated perimetry (SAP). For the macular region, dense testing grids, such as the standard 10-2, are used. The 10-2 spans the central 10° from fixation with a spacing of 2° between test locations. There is some evidence to suggest that these grids are more sensitive to early glaucoma damage in that region when compared to less dense testing grids, such as the 24-2 test pattern.^4^^,^^5^

Combining structural and functional information should further improve the identification of glaucomatous macular damage and the detection of its progression. Moreover, studying the relationship between the two measurements offers useful insights into the kinetics and pathophysiology of RGC loss and dysfunction in glaucoma.^6^^,^^7^ Models seeking to match structural and functional data to histology measurements of RGC density have been used to explore this relationship.^8^^–^^11^ Recently, a method proposed by Raza and Hood^12^ has been used to convert the GCL thickness into RGC density to investigate the relationship between RGC number and SAP sensitivity^13^^,^^14^ in healthy subjects and glaucoma patients.

The unique features of the inner retina in the macular region need to be considered when comparing structural and functional measurements. The most significant of these is the radial displacement of RGCs from the fovea so that RGCs receiving a stimulus in the parafoveal region are displaced toward the periphery with respect to the location of their corresponding photoreceptors.^15^^–^^17^ RGCs are connected to the corresponding photoreceptors via Henle's fibers, which have an oblique pathway in the parafoveal region. This displacement diminishes with eccentricity, becoming minor at around 10 visual degrees from the fovea.^15^^,^^18^ Different numerical models, based on, or verified by, histologic measurements of Henle's fibers have been proposed to account for this displacement.^15^^,^^18^^–^^20^ The most widely used of these models is the one proposed by Drasdo et al.^15^ This model is valuable in the context of structure-function analyses because the displacement calculation requires equivalence between the cumulative number of RGC receptive fields (RGC-RF), estimated through psychophysical measurements, and the number of RGC bodies, estimated through histology.^16^ Theoretically, when applied correctly, this model would allow a one-to-one correspondence between the number of RGCs, estimated from structural maps, and psychophysical measurements, estimated from SAP sensitivity (which depends on the number of RGC-RFs stimulated during the test).

Although widely used, the implementation of the model reported by Drasdo et al.^15^ is not straightforward. For example, Drasdo et al. only reported numerical calculations for the four principal meridians and the average displacement, in microns.^15^ Therefore, a method to generalize to any arbitrary meridian has not been available. A second example is that the schematic eye used by Drasdo et al.^15^^,^^21^ to convert visual degrees to millimeters of retina assumes a spherical shape for the retina of a certain radius. However, that radius is not the same as that assumed by Curcio and Allen^16^ in their published histology map of RGC density. Moreover, the radius of the sphere should be adjusted for the axial length (AL), when this is available. However, Drasdo et al.^15^^,^^21^ only provided average displacement values regardless of axial length. A third example is that, in many cases, a simple displacement of the stimulus centers was applied.^7^^,^^13^^,^^22^ However, the displacement should be applied to the perimeter of the stimulus, so that different points of the stimulus edge are independently displaced radially outward according to the model. For example, in the parafoveal region, the stimulus edge nearer the fovea is displaced further than the stimulus edge further from the fovea.^14^^,^^23^ This is especially important when RGC counts are involved, because small differences in the area used for calculations can result in large differences in the counts.

The objective of our work was to develop a revised version of the displacement model for any retinal location and with a customizable schematic eye, to account for variations in AL; determine the correct displacement model for circular perimetric stimulus (covering RGC-RFs) to corresponding RGC location. Moreover, we developed a web application to allow researchers to apply the revised model to their own structure-function data in an attempt to improve the comparability of findings from different research groups.

## Methods

### Datasets

For our analyses we used two datasets. The first (Dataset 1) was a collection of macular volume scans collected for the Northern Ireland Sensory Ageing (NISA) study (https://clinicaltrials.gov/ct2/show/NCT02788695), which originated from a population based aging cohort (NICOLA study https://www.qub.ac.uk/sites/NICOLA/) conducted in Belfast at Queen's University, Belfast. Scans were acquired with a Spectralis SD-OCT (Heidelberg Engineering, Heidelberg, Germany) comprised 61 horizontal B-scans centered on the fovea (ART 9, 30° × 25° with a fixed 7° rotation, counterclockwise for right eyes, clockwise for left eyes). In this dataset, 417/726 scans were classified as having a healthy outer retina by two graders. In 299 of these eyes, *AL* was measured using a Lenstar LS 900 Biometer (Haag-Streit AG, Switzerland). These scans were further screened by an ophthalmologist (GM) for pathologic changes of the inner retina. Seventeen scans were excluded because of poor quality that prevented a clear identification of the inner retinal layers or the Bruch's membrane within 15° from the fovea, 13 scans had local thinning that could be attributed either to glaucoma or local ischemia and four were excluded for vitreoretinal alterations. The segmentation of the retinal layers was checked and manually corrected where necessary leaving 265 scans for analysis. This dataset was used exclusively to extract metrics on the shape of the GCL profile. No thickness values were measured. The median [interquartile range] quality index (QI) was 30.6 [28.98, 32.26] dB.

The second dataset (Dataset 2) was a collection of SD-OCT scans acquired for a cross-sectional study on structure-function relationship in the healthy macula. The study was approved by the ethical committee Comitato Etico Milano Area 1 (code OCU_SSSF) and the data collection took place at the eye clinic at San Paolo Hospital (University of Milan) in Milan, Italy. The dataset included 28 macular scans from 28 subjects collected with a Spectralis SD-OCT and composed of 121 B-scans, centered on the fovea (ART 9, 25° × 30°, oriented vertically). *AL* was measured using an IOL-Master V3 A-scan (Zeiss Meditec, Dublin, CA, USA). The subjects had no known or detectable ocular disease and younger than 40 (range 23–37) years, to match the age range of the histological dataset collected by Curcio and Allen^16^ (see next section). Descriptive statistics for the two datasets are given in Table 1. Best corrected visual acuity in Dataset 2 was 0.00 LogMar for all subjects and was not measured further. All data collections were performed in agreement with the declaration of Helsinki after explicit written consent from the participants. All scans were of good quality and none was excluded (QI = 26.47 [25.36–27.47] dB).

**Table 1.** Descriptive Statistics for Relevant Variables in the Two Datasets

	Dataset 1 (*N* = 265)	Dataset 2 (*N* = 28)
**Median [Interquartile range]**		
Age (years)	58 [63, 68]	28 [26, 31]
Male:Female	134:131	15:13
BCVA (Letters)	89 [85, 92]	—
Spherical equivalent (Diopters)	0.5 [−0.5, 1.5]	−1.19 [−3.50, 0]
Axial length (*mm*)	22.94 [23.67, 24.34]	24.50 [23.89, 25.02]
Average macular GCL thickness (*µm*)^*^	33.34 [31.36, 35.39]	35.73 [33.89, 37.64]

BCVA, Best Corrected Visual Acuity.

* Calculations performed on the whole thickness map within 3.5 *mm* from the fovea.

### Histology Map

The original model developed by Drasdo et al.^15^ used the histology map provided by Curcio and Allen.^16^ This reports the density of ganglion cells (cells/*mm*^2^) obtained from six retinas of five healthy subjects, aged 27 to 37 years (range), for a retinal sphere with a radius of 11.459 *mm*. Details are reported in Appendix A.

### Schematic Eye

**Figure 1.**
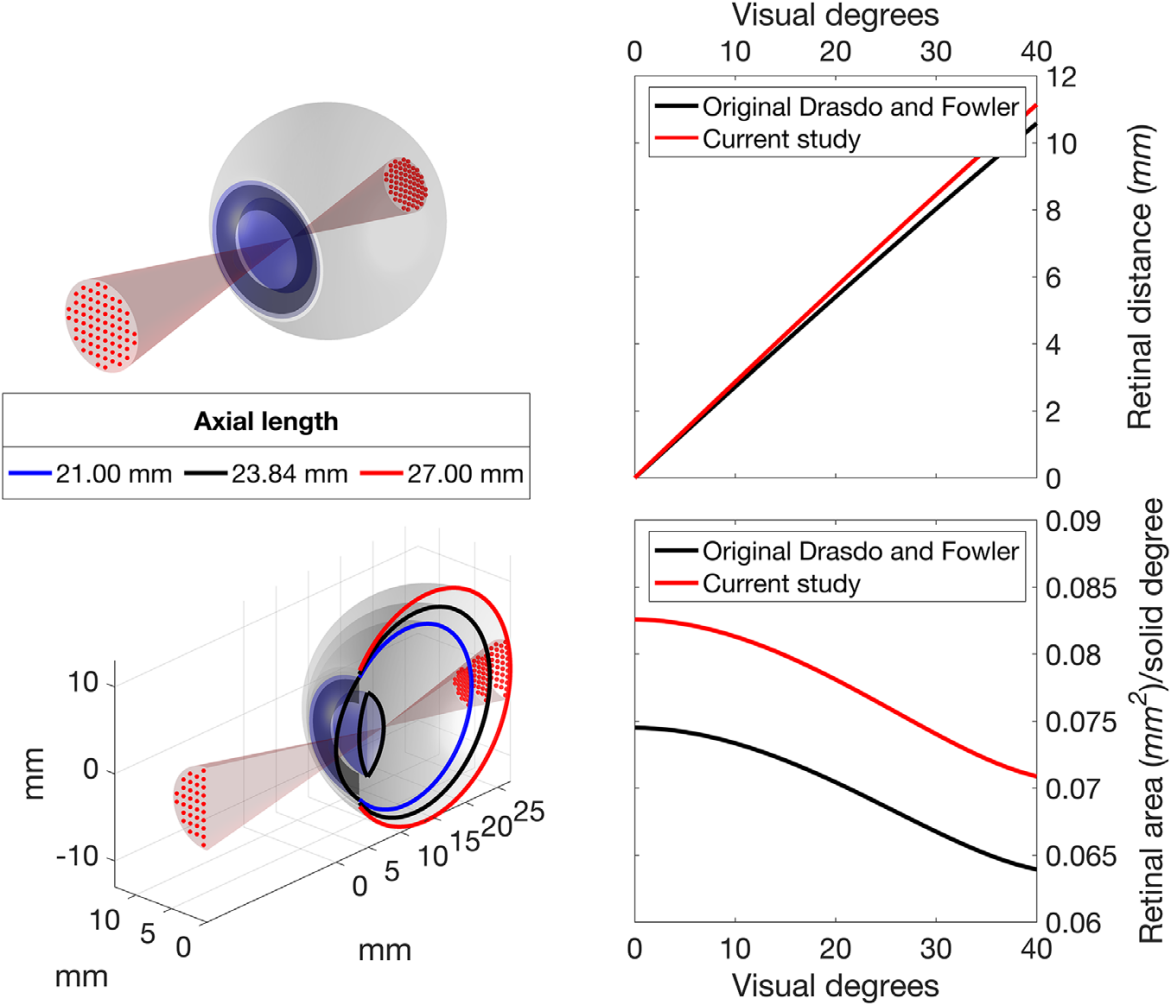
Rendering of the schematic eye, with a projection of a 10-2 grid (*red dots, top panel*). The bottom panel shows a cross-section of the same schematic eye with additional examples of short and long axial lengths. *Right panels* show the distance travelled on the retina per visual degree (*top*) and the mm^2^/solid degree ratio at various retinal eccentricities (*bottom*). The latter represents the ratio between retinal areas in mm^2^ to visual degrees^2^. The curved relationship with eccentricity is a consequence of the nonlinear projection obtained by numerical calculations of ray tracing through the cornea, lens and vitreous, which varies with visual angle. It is important to acknowledge that the relationship between retinal *mm* and degrees of visual angle is also not linear. In *black*, the curves obtained from the original schematic eye described by Drasdo and Fowler^21^ (in *black*). In *red*, the results of the schematic eye used in this study.

The schematic eye used in this work replicated the one described by Drasdo and Fowler^21^ and later used by Drasdo et al.^15^ for their displacement model. We used numerical ray tracing through the schematic eye to calculate the correspondence between visual degrees and *mm*, and solid visual degrees and *mm*^2^, on the retina. The data to build the schematic eye was derived from the table reported in the original article.^21^ Importantly, this approach aligns with the original methodology unlike that applied in previous studies.^18^^,^^19^ Note the radius of the retinal sphere has been changed to match the one used for the histology map (*r_0_* = 11.459 *mm*, originally 11.06 *mm* in Drasdo and Fowler).^21^ The distance between the center of the retinal sphere and the corneal vertex has also been scaled proportionally (*c_0_* = 12.38 *mm*, originally 11.95 *mm*). Therefore the default AL (AL_0_) of our schematic eye was 23.84 *mm* (originally 23.01 *mm*). These changes had a small impact on the degrees-to-*mm* conversion, but a more important effect of the *mm*^2^/solid degree ratio (Fig. 1). The schematic eye was coded in Matlab (The MathWorks, Natick, MA, USA). Additional details are reported in Appendix B.

### Scaling of Eye Structures and Cell Density

A customized displacement model must account for how retinal structures scale with *AL*, especially the size of the displacement area (see later) and the planar density of ganglion cells derived from histology. The assumption of a spherical shape for the retina for all ALs is prone to the adoption of a global expansion model. In this model, the planar RGC density would scale inversely with the square of the retinal radius, whereas the radius of the displacement zone (*r_DZ_* = 4.034 *mm* in the original paper)^15^ would scale linearly with the retinal radius. The two scaling equations, where *r* is the retinal radius corresponding to a given AL, are given as:

(1) $$r_{DZ}\left(AL\right)=\;4.034\;*\;\left(\frac{r}{r_{0}}\right),$$

(2) $$RGC\;Density\left(AL\right)=\;RGC\;Density\left(AL_{0}\right)*{\left(\frac{r_{0}}{r}\right)}^{2}.$$

An alternative model to the assumption of global expansion is “equatorial stretch,” where the posterior pole is simply moved further away from the corneal vertex with no change in the relative size of the retinal structures. Although the actual expansion process in myopia is likely to be a mixture of the two phenomena,^24^^–^^26^ psychophysical evidence suggest that a global expansion model is a reasonable approximation for most axial lengths.^27^^–^^30^ Global expansion is also implied in the RGC-RF model proposed by Drasdo et al.,^15^ which assumes a constant density of RFs per solid visual degree (see later).

A global expansion model also implies that the amount of radial RGC displacement, when measured in *mm* on the retina, should increase with axial length. This is a consequence of the stretching of the retinal tissue and Henle's fibers with increasing eye size. Although direct evidence of this is not yet available, indirect confirmation can be obtained by observing how the GCL profile scales with axial length in healthy eyes. To explore this, we used the 265 macular volume scans from Dataset 1 and identified the maximum GCL thickness peak for several meridians, centered on the anatomic fovea (Fig. 3). An ellipse was then fitted through a least squares method to the locations of the peaks. We then measured the length of the major and minor axes of the ellipse. All measurements were corrected for ocular magnification using the schematic eye defined in the previous section. The relationship between the length of the ellipse axes and the axial length was explored through linear regression. The ellipse dimensions were also predicted for an exact scaling with axial length, assuming a global expansion, by multiplying the ellipse dimensions predicted from the linear regression at *AL_0_* by the same scaling factor used for the *r_DZ_* (geometric scaling model). The goodness-of-fit of the linear regression and the geometric scaling model were compared using the mean absolute error (MAE), calculated for each model as the average of the absolute residuals.

### OCT Data Processing

All OCT data were exported as RAW files (.vol) using the Heidelberg Eye Explorer. The files were then imported in Matlab using a custom routine. The segmentations were then used to generate thickness maps for the whole retina and the GCL. The maps were interpolated and smoothed to match the size of the reference infrared fundus image (768 × 768 pixels, 30° × 30° field of view), padding with zeros where the OCT data were missing, i.e., outside the scanning pattern. The interpolation was performed using a thin plate spline (*tpaps* function in Matlab) with anisotropic smoothing parameters, so that smoothing was stronger across B-scans than within a B-scan. The fovea was automatically identified through a template matching. Correct detection was confirmed through visual inspection.

### Conversion of GCL Thickness Maps into Estimated RGC Counts

We used the method proposed by Raza and Hood^12^ to convert the OCT GCL thickness maps into customized estimates of RGC density and applied this to Dataset 2. In brief, the histology map was divided point-by-point by an average healthy GCL thickness map (768 × 768 pixels), obtained as the average from all eyes in Dataset 2, after aligning the fovea and the position of the optic nerve head (ONH). This yielded a volumetric density map (RGC/*mm*^3^). The map can then be multiplied point-by-point by a GCL thickness map from a new subject to obtain a customized RGC density map (RGC/*mm*^2^). We accounted for AL by applying a magnification correction to the GCL macular volume scans and by rescaling the histology density map according to the global expansion model given by Equation (2).

### Displacement Model

For the displacement model, we followed the same methodology proposed by Drasdo et al.^15^ in their original article. The first step was to calculate the RGC-RF density along a specific meridian obtained from a generic model based on psychophysical measurements, the derivation of which is described in detail in the original article. The final formula, where *e* is the eccentricity in visual degrees, *D_gcrf_* is the density of RGC-RF (number/solid degree), *R_v_* = 0.011785 and *R_o_* = 0.008333 and *k* is a parameter that depends on eccentricity (see Appendix C), is given as follows:

(3) $$D_{gcrf}\left(e\right)=\;\frac{k*\left(1.12+0.0273*e\right)}{1.155*\left({\left(R_{v}\left(1+\;e/E_{2v}\right)\right)}^{2}-\;{\left(R_{o}\left(1+\;e/20\right)\right)}^{2}\right)}.$$

The parameter *E_2v_* in Equation (3) was used by Drasdo et al.^15^ to scale the RGC-RF for each principal meridian. A key objective of our new approach was to determine its value for any arbitrary meridian. Similarly to Drasdo et al.,^15^ we performed a numerical optimization of this parameter by simply requiring that the total counts of RGC-RF and RGC bodies are equal within the maximum displacement zone (*DZ*). From Drasdo et al.,^15^ the *DZ* ends at 4.034 *mm* from the fovea and is assumed symmetric. This value was used for *AL_0_* and was scaled proportionally with the retinal radius for different axial lengths, as previously explained. The displacement is finally computed as the difference between the eccentricities at which the cumulative count of RGC bodies (*C_gcb_*) and the cumulative count of RGC-RF (*C_gcrf_*) are equal. Additional details are reported in Appendix C.

### Displacement of Perimetric Stimuli

**Figure 2.**
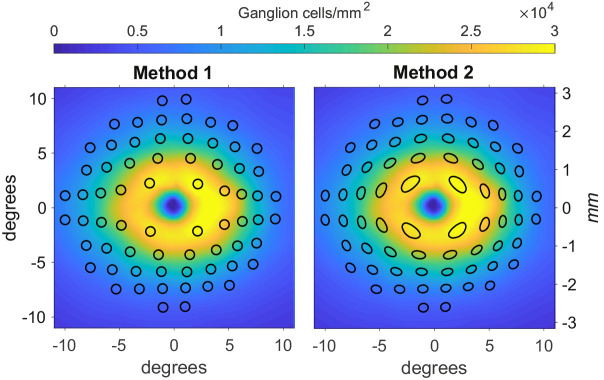
Representations of the two candidate methods to apply the displacement to perimetric stimuli. Method 1 is the one applied by Yoshioka et al.^13^. Method 2 is the one proposed in this article. The color map represents the histology density at AL_0_ for both graphs. The vertical axes are reported both in *mm* and visual degrees. The *black shapes* represent the areas tested by a 10-2 perimetric test, displaced with the two methods. In both images we are displaying the results of the application of the two methods on Goldmann IV stimuli, assumed circular and arranged in a regular 10-2 grid.

We compared two methods of applying the Drasdo model to perimetric stimuli. The first commonly-applied method ^13^ (Method 1) consisted of a simple displacement of the center of the stimuli, without any changes to its shape (Fig. 2, left panel). In the alternative method (Method 2), the circumference of the stimulus (approximated with 72 points around the stimulus edge) is displaced according to the Drasdo model; this results in distorted stimulus shapes in the parafoveal region (Fig. 2, right panel). We tested the accuracy of each method by requiring consistency under the Drasdo model. In fact, the model calculates the displacement by equating the number of expected RGC-RF and the number of RGC bodies at any given eccentricity (in a healthy eye). Therefore the estimated number of RGC-RF within a given stimulus area should match the number of RGC bodies within the displaced stimulus on the structural map, besides some minimal discrepancy due to approximation errors in the numerical calculations. For our calculations, we used a 10-2 grid and calculated the number of RGC-RF and cellular bodies for all conventional Goldman sizes, from I to V. The RGC-RF density function can change for each meridian. Hence, we generated a dense map with the same resolution as the structural map and used binary masks to calculate the number of RGC-RFs within each stimulus size. The same methodology was applied to the displaced stimuli on the structural map. The resolution used for our calculations was 2.2 µm (0.008°) for the histology map and 0.0391° for the structural maps in Dataset 2 (the maximum resolution of the Spectralis SD-OCT). This mainly affected the precision of the binary masks, which is important for small stimulus sizes. For the first analysis, we aimed for a very precise quantification to test the theoretical validity of the two methods. For the second analysis, we used a resolution that is likely to be applied for real data as a compromise between precision and speed of calculation.

### Development of the Web App

A web application (App) was developed using the Shiny library^31^ for R^32^ (R Foundation for Statistical Computing, Vienna, Austria). It allows the visualization of the schematic eye and the calculation for the RGC displacement. It can also import structural maps and provide calculations for different stimulus sizes. Finally, it can also be used for batch processing of a whole dataset. The App is freely available at https://relayer.online/drasdo, with detailed explanations on its use.

The Matlab codes for the schematic eye and the displacement model were translated in R. For faster computational execution, the displacement was pre-calculated for different *ALs* (from 18 to 35 *mm*, at 0.5 *mm* intervals). The planar displacement maps were calculated out to 7.5 *mm* from the fovea, at 0.05 *mm* intervals, then organized in a dense three-dimensional array. The displacement values are then obtained via linear interpolation of the array.

## Results

### Scaling of Eye Structures with Axial Length

**Figure 3.**
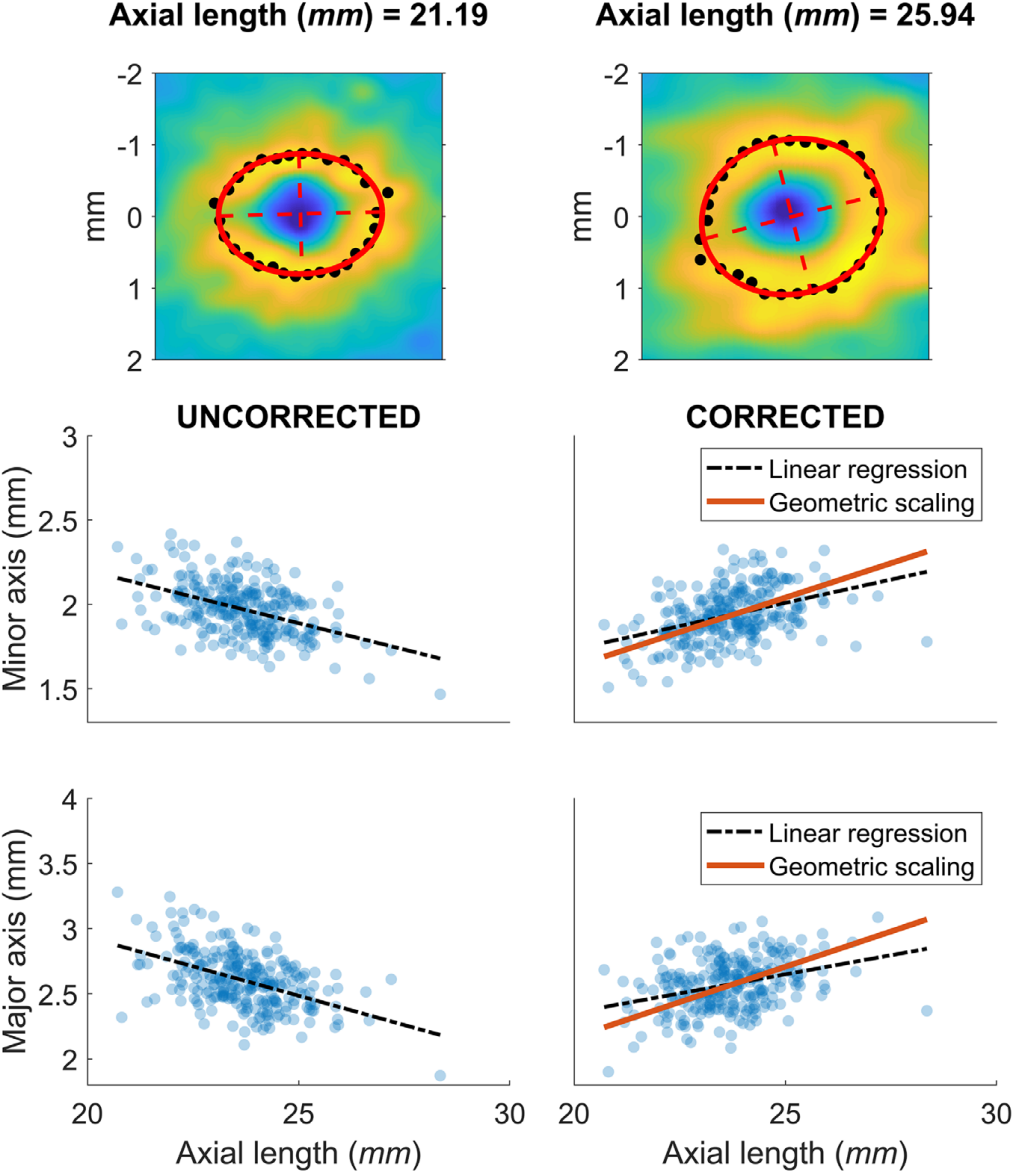
Scaling of ocular structures with axial lengths. *Top panels* show two examples of the calculation for the descriptive ellipses (in *red*), as described in the Methods. The *black points* identify the peaks in the GCL profile used for the fitting. *Lower panels* show the measurements of the major and minor axis of the ellipses before (*left*) and after (*right*) correction for ocular magnification.

The calculations were performed on the eyes from Dataset 1. Both axes of the ellipses correlated negatively with *AL* before magnification correction (major axis MAE = 0.149 *mm*, *P* < 0.001; minor axis MAE = 0.111 *mm*, *P* < 0.001) and positively after magnification correction (major axis MAE = 0.148 *mm*, *P* < 0.001; minor axis MAE = 0.112 *mm*, *P* < 0.001). A geometric scaling model offered a very similar fit (major axis MAE = 0.175 *mm*; minor axis MAE = 0.131 *mm*). The results are presented in Figure 3.

### Variability of Displacement with Axial Length

**Figure 4.**
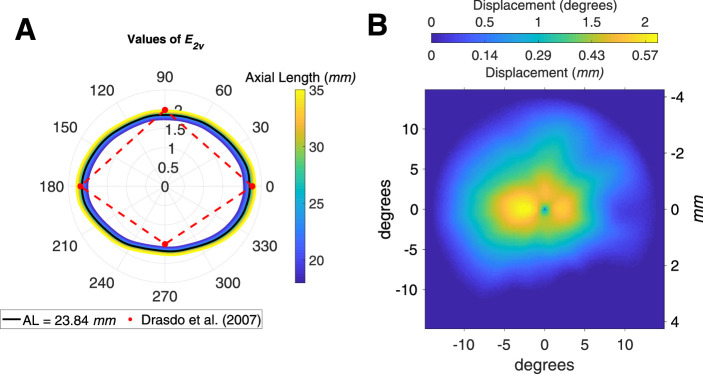
(A) Polar plot of the results of the fitting process for the parameter *E_2v_*. N, nasal; S, superior; T, temporal; I, inferior. (B) Displacement map calculated in degrees and *mm* at *AL_0_*.

Results of the fitting process for the parameter *E_2v_* are reported in Figure 4A. Values are very similar to those reported by Drasdo et al.^15^ for the principal meridians for all considered *ALs*. The systematic change with axial length was small (Fig. 4A). The displacement is constant for all axial lengths when measured in degrees, as a consequence of the global expansion mechanism assumed by the model. Figure 4 B shows the displacement in degrees and in *mm* for *AL_0_*. The values in *mm* are very similar to the average displacement reported by Drasdo et al.^15^

### Displacement of Perimetric Stimuli

**Figure 5.**
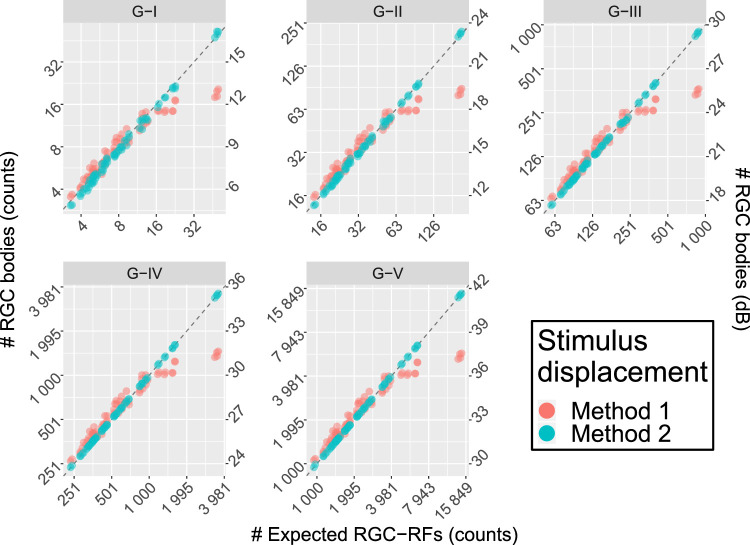
Comparison of the results between the two displacement methods for perimetric stimuli. Method 1 is the one used by Yoshioka et al.^13^. Method 2 is the one proposed in this article. The horizontal axis reports the expected RGC-RF counts, calculated from the model proposed by Drasdo et al.,^15^ (Equation (3)) and do not represent real subject data. The vertical axis reports the structural measurements from the RGC map both as counts (*left axis*) and in dB (*right axis*). The latter is meant for easier comparison with the results in Yoshioka et al.^13^. Only Method 2 yields correct estimates in the parafoveal region (higher counts). The *dashed line* represents the ideal line of equivalence.

**Figure 6.**
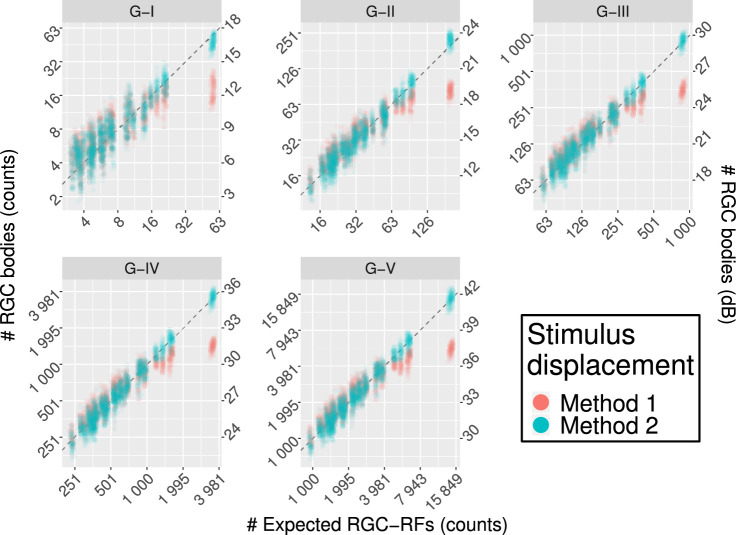
Comparison of the results between the two displacement methods for perimetric stimuli for real structural data. Method 1 is the one used by Yoshioka et al.^13^ Method 2 is the one proposed in this article. The *horizontal axis* reports the expected RGC-RF counts, calculated from the model proposed by Drasdo et al.,^15^ (Equation (3), adjusted for *AL*). The vertical axis reports the structural measurements from the RGC map both as counts (*left axis*) and in dB (*right axis*). The latter is meant for easier comparison with the results in Yoshioka et al.^13^. Only Method 2 yields correct estimates in the parafoveal region (higher counts). The *dashed line* represents the ideal line of equivalence.

Average density values (per solid degree) at different eccentricities are reported in Table 2 (calculated from the counts for the G-IV stimulus size). Method 1 yielded substantial underestimation of the RGC body counts in the parafoveal region, where the displacement is largest, and a slight overestimation at larger eccentricities (Fig. 5 and Table 2). Conversely, Method 2 provided estimates that were very consistent with the expected number of RGC-RF. The slightly larger variability with a G-I stimulus was due to numerical approximation and completely disappears for larger stimulus sizes. When converted to dB units (Fig. 5), the calculations with Method 1 yield similar results to those reported by Yoshioka et al.^13^ for healthy subjects. The same method was applied to the healthy macular volume SD-OCT scans in Dataset 2 (Fig. 6 and Table 2). The results were very similar to those obtained with the RGC histology map.

**Table 2.** Density Data at Different Eccentricities of the 10-2 Grid, Derived From the Counts Reported in Figure 5 and Figure 6 for a G-IV Stimulus Size

			RGC Body Density	
	Eccentricity (Degrees)	RGC-RF Density	Method 1	Method 2
RGC histology map	1.41	5969 (157)	2392 (105)	5998 (173)
	3.16	2435 (307)	1868 (165)	2433 (304)
	4.24	1609 (58)	1551 (143)	1596 (62)
	5.1	1288 (210)	1332 (234)	1292 (212)
	5.83	1026 (90)	1097 (120)	1028 (90)
	7.07	783 (116)	861 (154)	785 (116)
	7.62	689 (91)	753 (94)	690 (91)
	8.6	560 (39)	609 (40)	560 (39)
	9.06	540 (102)	589 (114)	542 (102)
Database 2	1.41	5975 (145)	2424 (211)	6085 (566)
	3.16	2445 (291)	1894 (243)	2476 (388)
	4.24	1623 (54)	1520 (191)	1576 (180)
	5.1	1295 (199)	1343 (268)	1306 (269)
	5.83	1040 (87)	1105 (159)	1035 (152)
	7.07	790 (112)	859 (188)	782 (168)
	7.62	696 (87)	753 (132)	691 (127)
	8.6	568 (38)	614 (92)	563 (90)
	9.06	541 (97)	591 (146)	545 (133)

Values are reported as mean (SD). For the RGC histology map, the SD refers to different locations with the same eccentricity.

## Discussion

In our work we revisited the RGC displacement model proposed by Drasdo et al.^15^ and studied its application to perimetric data to yield consistent structure-function measurements. We also developed a web App to make our methodology easily available for other researchers, in the hope to standardize this essential aspect of structure-function analyses.

Our implementation generalized the displacement model to any arbitrary meridian. Compared to other models ^19^^,^^20^ we imposed weaker constraints on the symmetry of the displacement. The model proposed by Sjostrand et al.^20^ used histological measurements to derive an even displacement around the fovea. Watson ^19^ followed an approach similar to Drasdo et al.^15^ but used a different equation for the RGC-RFs and extended his calculations to arbitrary meridians by assuming an elliptical symmetry around the fovea. In contrast, our approach, as in the original article, only assumes the maximum displacement to be the same for all meridians in the fitting process. However, as shown in Figure 4B, such an assumption does not prevent the displacement from adapting to the measured distributions of RGC cells provided by histology. Importantly, the effective RGC displacement region extends to smaller eccentricities in the inferior retina. A similar approach for generalization of the Drasdo model has been proposed by Turpin et al.^18^ Our results were in general agreement; they also showed a smaller extent of the displacement inferiorly compared to other regions. However, the displacement for the parafoveal locations was smaller in our calculations and in good agreement with the average displacement calculated by Drasdo et al.^15^ In addition to previous work, we implemented a numerical ray tracing model of the schematic eye used by Drasdo and Fowler^21^ to convert between visual degrees and distances on the retinal sphere. This allowed us to adapt the model so that the retinal sphere corresponded to the one used for the retinal histology map built by Curcio and Allen.^16^^,^^33^ This is crucial to obtain consistent calculations, because the Drasdo model is based on that map. The implementation of the numerical model also allowed us to customize the conversion and the RGC density map based on the axial length. In this study, we assumed a global expansion model, scaling the linear structures with the radius of the retinal sphere and the density with the squared radius; this has been shown to be a good approximation by psychophysical examinations.^27^^–^^30^ Additionally, we confirmed this by observing how the structure of the inner retina scales with axial length using a large dataset of SD-OCT data (Fig. 3). We found that geometric scaling for axial length fitted the observed data adjusted for ocular magnification. Under this assumption, the displacement is conveniently equivalent for all axial lengths when calculated in degrees of visual angle. However, competing models have been proposed for eye growth in myopia and an elliptical growth model, combining equatorial stretching and global expansion, seems to be the most realistic from anatomic studies.^24^^–^^26^ One advantage of our numerical implementation of the schematic eye is that it can be easily adapted to accommodate for different types of expansion models. One major limitation of our structural dataset was the lack of extreme axial lengths. Determining the optimal expansion model with a stratified data collection of structural and functional data will be the objective of future work.

Our work was novel because it considered two different methods of applying the displacement to perimetric stimuli in structure function analyses. We showed that simply displacing the stimulus center (Method 1) does not provide estimates of RGC-RF counts within perimetric stimuli consistent with the counts expected from the Drasdo model. Instead, each point on the edge of the perimetric stimulus needs to be displaced independently (Method 2), resulting in distorted, ovoidal shapes. We were able to verify the validity of this approach by requiring that the RGC counts within a given displaced stimulus from the histology map be consistent with the expected RGC-RF counts assumed by the Drasdo model (Equation (3)). Only Method 2 yielded correct estimates (Fig. 5). We then verified that these results hold when the two methods are applied to structural data from young healthy subjects (Fig. 6). The increase in variability in this latter analysis was due both to intrasubject differences in the structural data and to the fact that the calculations were limited to the resolution of the structural maps, as explained in the Methods. Method 1 is similar to what was used by Yoshihoka et al.^13^ Unfortunately, those authors did not report tabulated RGC counts or estimated density. Nevertheless, the graphs reported in Figure 2 of their article^13^ clearly show counts that, in healthy subjects, are compatible with the results of Method 1. For example, the largest RGC counts for a G-III stimulus were approximately 25.6 dB, very similar to our results in Figures 5 and 6 for Method 1 (25.5 dB). In turn, this was crucially less than half than that derived from Method 2 (29.5 dB) and the expected RGC-RF count from Equation (3) (29.4 dB). Moreover, the RGC-RF density derived from Method 1 for the smallest eccentricity (1.41°, Table 2), when substituted into Equation (3) to derive the corresponding visual acuity (with *E_2v_* = 2), yields a value of 16.5 cycles/degree, unreasonably low for this eccentricity.^15^ In contrast, Method 2 yields 24.5 cycles/degree, much closer to the predicted 24.9 cycles/degree^15^ and compatible with the literature.^34^ These discrepancies might also be due to the fact that Yoshioka et al.^13^ provided age-corrected structural and functional measurements at 64.5 years of age. However, the boxplots in the supplementary material for the same article show minimal changes between age-corrected and raw thickness values, too low to justify such a large difference.

Our findings are of particular importance for the interpretation of previously published results. To the extent of our knowledge, Method 2 has only been applied twice in the literature.^14^^,^^23^ Moreover, the actual methodology to implement the Drasdo model has been rarely reported. In many cases, the displacement appears to be symmetrical around the fovea.^7^^,^^13^^,^^22^^,^^35^^–^^38^ This likely indicates an application of the average displacement profile presented in the graph from Figure 6 in the original paper by Drasdo et al.,^15^ using a fixed degrees to *mm* conversion. Although this might be satisfactory for some simple correlation analyses, disregarding the asymmetric nature of the displacement limits studies where a more detailed structure-function relationship is sought. For instance this is important when the development of a neural model of functional response is the main goal of the research.^13^ In fact, as shown by our results in Figure 6, a high degree of consistency with the calculations can be achieved, especially considering that, like the Drasdo displacement model, the method to estimate the number of RGC cell bodies from structural measurements^12^ is also based on the structural map produced by Curcio and Allen.^16^

**Figure 7.**
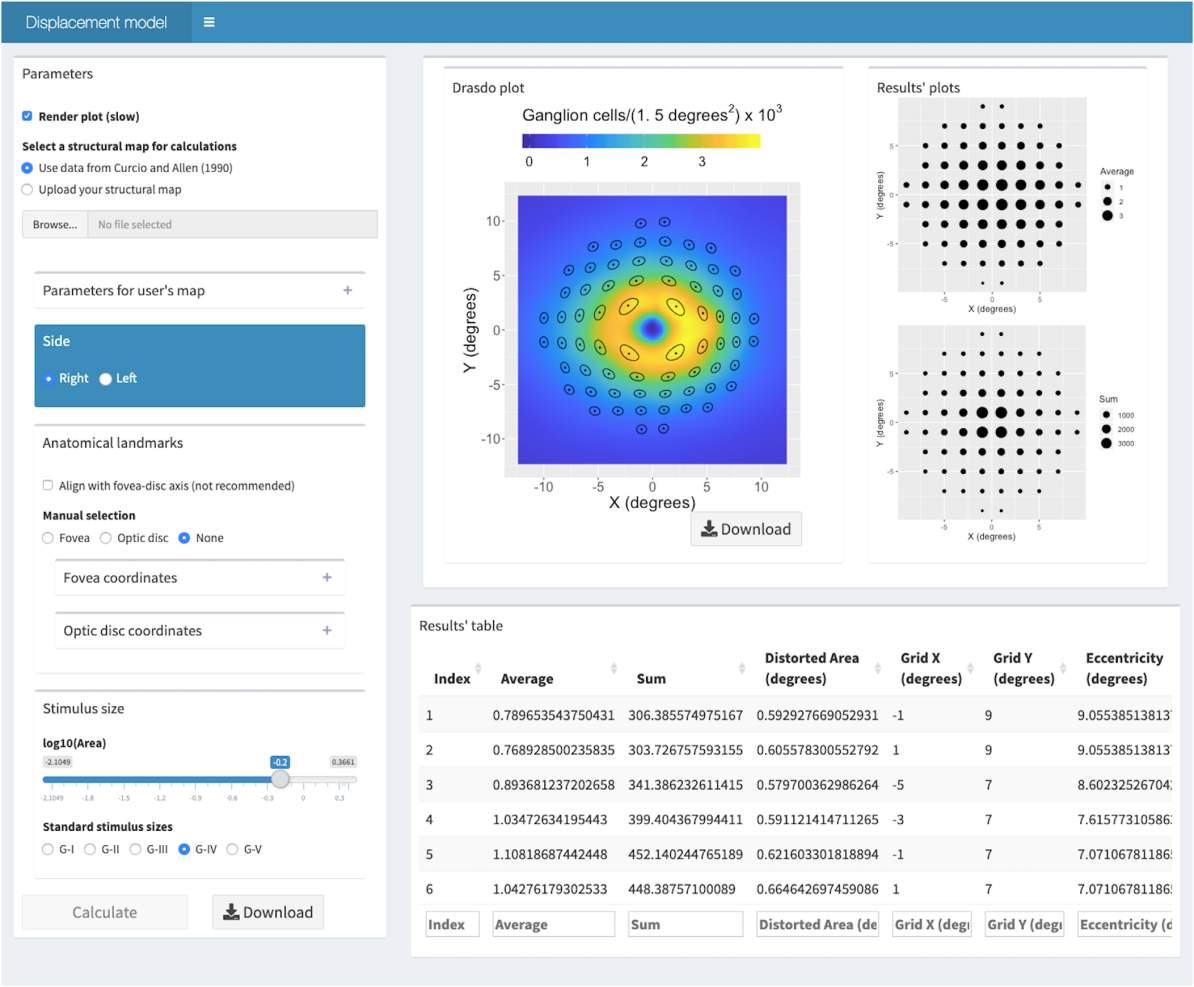
Screenshot of the second screen from the web App. It presents the results for the schematic eye at *AL_0_*, for a G-IV stimulus size and a 10-2 perimetric grid using the histological dataset by Curcio and Allen as a structural map. The Results table extends beyond what is visible on the screen and can be easily downloaded.

To encourage translation, we have made our methodology available for researchers in a free user-friendly web App (Fig. 7, https://relayer.online/drasdo). The App allows for a straightforward and customizable application of the displacement model for different axial lengths, any perimetric grids, varied stimulus sizes and structural maps. Graphical outputs are designed to provide the researcher with tools to scrutinize the steps in the process. Batch analysis can also be done on large datasets. The App will be updated with future development of the methodology; for example, when a more comprehensive expansion model is developed.

## Figures and Tables

**Figure A1.**
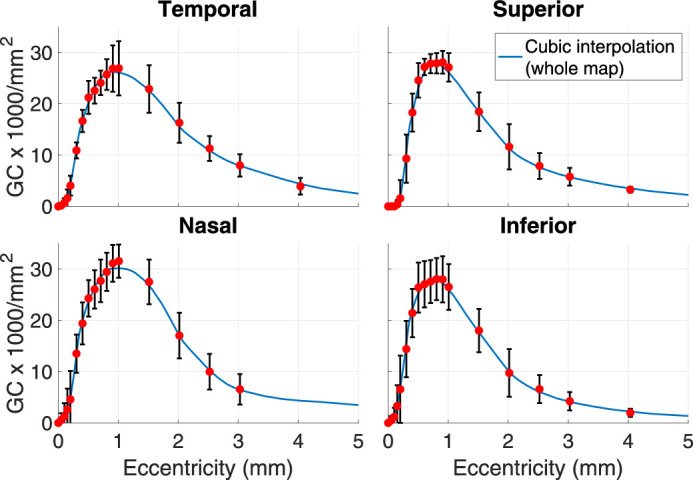
Comparison of the cubic interpolation from the whole spherical map and the raw averages (*red dots*) and standard deviations (*black vertical bars*) reported by Curcio and Allen.^16^

**Figure A2.**
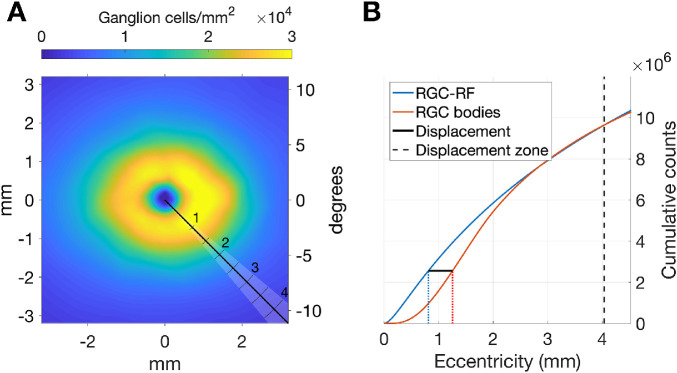
Example of how the displacement is computed along a specific meridian. (A) The *black line* indicates the meridian analyzed. The *shaded area* highlights the radial sector. The *ticks* indicate the distance in *mm*. The color map represents the RGC histology density map. (B) The displacement is calculated as the distance (*black solid line*) between the two eccentricities at which the cumulative counts of RGC-RF (*blue solid line*) and the RGC bodies (*red solid line*) are equal. The *dashed vertical line* indicates the eccentricity of the maximum displacement zone. The fitting process requires that the two cumulative counts are the same at this eccentricity.

## Appendix A

As described in a separate article,^33^ the data were recorded by Curcio and Allen using spherical coordinates, reporting co-latitude (or retinal eccentricity) and longitude (or retinal meridian) in degrees. Eccentricity is calculated from the fovea, located at (0, 0). One degree of retinal eccentricity equals 2πr/360, where r is the radius of the assumed retinal sphere. From the header of the text file containing the tabulated data (https://research-materials.christineacurcio.com/), the assumed retinal sphere for the map is 11.459 *mm*. The map is built for a left eye, with the ONH at 20° eccentricity on the horizontal meridian (180° longitude). After conversion to *mm*, a continuous histology map was obtained through cubic interpolation. Figure A1 shows the correspondence between the interpolated values and the original average counts (with standard deviations) provided by Curcio and Allen (https://research-materials.christineacurcio.com/) for the four principal meridians. The map was converted into a right eye by inverting the horizontal coordinates.

## Appendix B

The data for the schematic eye are reported in Table A1. The ray tracing allows precise calculations for different *ALs*, which can be achieved by proportionally changing the radius and the center location of the retinal sphere (Fig. 1). The anterior part of the schematic eye is left unchanged, so that the nodal point remains the same for all *ALs*. The numerical estimate for the nodal point was obtained by averaging of 670 ray-tracings, with an angle of incidence between 0.1° and 67° with respect to the optic axis. The nodal point for each incident ray was derived numerically by minimizing the absolute difference between the angle of the incident and the refracted ray (after the posterior face of the lens) with respect to the optic axis. The resulting estimate was 6.93 *mm*, which is very close to the value of 6.95 *mm* reported by Drasdo and Fowler.^21^ The schematic eye was assumed to be radially symmetric around the optic axis. The same schematic eye was used to correct for ocular magnification in the SD-OCT macular volume scans. In their original article, Drasdo and Fowler did not clarify what point they used as a reference to calculate the visual angle. In accordance with the guidelines from the Imaging and Perimetry Society,^39^ we used the nodal point of the schematic eye.

**Table A1.** Data for the Schematic Eye Used in this Paper Along with the Original Values from Drasdo and Fowler^21^

	Current Study	Original Values^21^
**Distance from corneal vertex (*mm*)**		
Retina (Axial length)	**23.8401**	**23.0100**
First nodal point	**6.930**	**6.950**
Anterior lens surface	3.600	3.600
Posterior lens surface	**7.375**	**Missing**
Centre of retinal sphere	**12.381**	**11.950**
**Radii of curvature (*mm*)**		
Retina	**11.459**	**11.060**
Anterior lens surface	10.000	10.000
Posterior lens surface	6.000	6.000
Apex of cornea	7.800	7.800
Eccentricity of corneal ellipse	0.500	0.500
**Refractive indices**		
Cornea, aqueous, vitreous	1.336	1.336
Lens	1.430	1.430

The differences are highlighted in bold. Missing = inferred from Figure 1 in the original article.

## Appendix C

The value of *k* in formula 3 also depends on eccentricity according to

(C.1) $$\begin{array}{ccc} k\left(e\right) & = & \;1+(1.004-0.007209\;*\;e+0.001694\;*\;e^{2}\\ & & -0.00003765\;*\;e^{3}{)}^{-2} \end{array}$$

and accounts for the change in the relative percentage of the ON and OFF midget RGC-RF with eccentricity, as reported by Drasdo et al.^15^ The *D_gcrf_* is converted into a density per *mm*^2^ with the conversion ratio derived from the schematic eye (Fig. 1), according to the axial length. The eccentricity in visual degrees is also converted into *mm* on the retina using the schematic eye. Then, an RGC-RF density profile from the fovea outward can be calculated and converted into cumulative counts for a circular sector centered at the fovea by

(C.2) $$C_{gcrf}\left(r\right)=\int_{0}^{r}\left[2\pi r\;*\;D_{gcrf}\left(r\right)\right]dr$$

where *r* is the eccentricity from the fovea in *mm* and *2πr* is a correction factor for the sector area increasing with eccentricity. The actual width of the sector considered acts only as a scaling factor and has no bearing on the results of the computation. When computed numerically, the integral is simply a cumulative summation for predefined steps in *r*. The same formula was used to compute the cumulative counts of RGC bodies (*C_gcb_*). In this case, the *D_gcrf_* is simply replaced by the density of RGC bodies (per *mm*^2^) obtained from the histology map (*D_gcb_*) along the same meridian, after correction for the size of the retinal sphere. The displacement is finally computed as the difference between the eccentricities at which *C_gcb_* and *C_gcrf_* are equal (Fig. A2). For each meridian, the displacement is zero beyond the first crossing point between the two cumulative curves. Therefore the actual displacement might involve a smaller area than the *DZ*.

## References

[1] Glen FC, Crabb DP, Smith ND, Burton R, Garway-Heath DF Do patients with glaucoma have difficulty recognizing faces? *Invest Ophthalmol Vis Sci*. 2012; 53: 3629–3637. 22511628 10.1167/iovs.11-8538

[2] Burton R, Smith ND, Crabb DP Eye movements and reading in glaucoma: observations on patients with advanced visual field loss. *Graefes Arch Clin Exp Ophthalmol*. 2014; 252: 1621–1630. 25074043 10.1007/s00417-014-2752-x

[3] Glen FC, Smith ND, Crabb DP Saccadic eye movements and face recognition performance in patients with central glaucomatous visual field defects. *Vis Res*. 2013; 82: 42–51. 23485426 10.1016/j.visres.2013.02.010

[4] De Moraes CG, Hood DC, Thenappan A, et al. 24-2 Visual Fields miss central defects shown on 10-2 tests in glaucoma suspects, ocular hypertensives, and early glaucoma. *Ophthalmology*. 2017; 124: 1449–1456. 28551166 10.1016/j.ophtha.2017.04.021 PMC5610609

[5] Grillo LM, Wang DL, Ramachandran R, et al. The 24-2 Visual Field Test misses central macular damage confirmed by the 10-2 Visual Field Test and Optical Coherence Tomography. *Transl Vis Sci Technol*. 2016; 5: 15. 10.1167/tvst.5.2.15 PMC4849532 27134774

[6] Hood DC, Kardon RH. A framework for comparing structural and functional measures of glaucomatous damage. *Progr Retinal Eye Res*. 2007; 26: 688–710. 10.1016/j.preteyeres.2007.08.001 PMC2110881 17889587

[7] Hood DC, Raza AS, de Moraes CG, Liebmann JM, Ritch R Glaucomatous damage of the macula. *Progr Retinal Eye Res*. 2013; 32: 1–21. 10.1016/j.preteyeres.2012.08.003 PMC3529818 22995953

[8] Garway-Heath DF, Caprioli J, Fitzke FW, Hitchings RA Scaling the hill of vision: the physiological relationship between light sensitivity and ganglion cell numbers. *Invest Ophthalmol Vis Sci*. 2000; 41: 1774–1782. 10845598

[9] Swanson WH, Felius J, Pan F Perimetric defects and ganglion cell damage: interpreting linear relations using a two-stage neural model. *Invest Ophthalmol Vis Sci*. 2004; 45: 466–472. 14744886 10.1167/iovs.03-0374

[10] Harwerth RS, Wheat JL, Fredette MJ, Anderson DR Linking structure and function in glaucoma. *Progr Retinal Eye Res*. 2010; 29: 249–271. 10.1016/j.preteyeres.2010.02.001 PMC2878911 20226873

[11] Anderson DR, Knighton RW Perimetry and acuity perimetry. In: SMPIKA (ed.), *Perspectives in Glaucoma*. New Jersey, Thorofare, NJ: Slack, Inc.; 1988: 59–70.

[12] Raza AS, Hood DC. Evaluation of the structure-function relationship in glaucoma using a novel method for estimating the number of retinal ganglion cells in the human retina. *Invest Ophthalmol Vis Sci*. 2015; 56: 5548–5556. 26305526 10.1167/iovs.14-16366 PMC4553929

[13] Yoshioka N, Zangerl B, Phu J, et al. Consistency of structure-function correlation between spatially scaled visual field stimuli and in vivo OCT ganglion cell counts. *Invest Ophthalmol Vis Sci*. 2018; 59: 1693–1703. 29610852 10.1167/iovs.17-23683

[14] Montesano G, Rossetti LM, Allegrini D, Romano MR, Crabb DP Improving visual field examination of the macula using structural information. *Transl Vis Sci Technol*. 2018; 7: 36. 30619656 10.1167/tvst.7.6.36 PMC6314223

[15] Drasdo N, Millican CL, Katholi CR, Curcio CA The length of Henle fibers in the human retina and a model of ganglion receptive field density in the visual field. *Vis Res*. 2007; 47: 2901–2911. 17320143 10.1016/j.visres.2007.01.007 PMC2077907

[16] Curcio CA, Allen KA. Topography of ganglion cells in human retina. *J Comp Neurol*. 1990; 300: 5–25. 2229487 10.1002/cne.903000103

[17] Lujan BJ, Roorda A, Knighton RW, Carroll J Revealing Henle's fiber layer using spectral domain optical coherence tomography. *Invest Ophthalmol Vis Sci*. 2011; 52: 1486–1492. 21071737 10.1167/iovs.10-5946 PMC3101665

[18] Turpin A, Chen S, Sepulveda JA, McKendrick AM Customizing structure-function displacements in the macula for individual differences. *Invest Ophthalmol Vis Sci*. 2015; 56: 5984–5989. 26393464 10.1167/iovs.15-17384

[19] Watson AB. A formula for human retinal ganglion cell receptive field density as a function of visual field location. *Journal of Vision*. 2014; 14: 15. 10.1167/14.7.15 24982468

[20] Sjostrand J, Popovic Z, Conradi N, Marshall J Morphometric study of the displacement of retinal ganglion cells subserving cones within the human fovea. *Graefes Arch Clin Exp Ophthalmol*. 1999; 237: 1014–1023. 10654171 10.1007/s004170050338

[21] Drasdo N, Fowler CW. Non-linear projection of the retinal image in a wide-angle schematic eye. *Br J Ophthalmol*. 1974; 58: 709–714. 4433482 10.1136/bjo.58.8.709 PMC1215006

[22] Miraftabi A, Amini N, Morales E, et al. Macular SD-OCT outcome measures: comparison of local structure-function relationships and dynamic range. *Invest Ophthalmol Vis Sci*. 2016; 57: 4815–4823. 27623336 10.1167/iovs.16-19648 PMC5024670

[23] Hirasawa K, Matsuura M, Fujino Y, et al. Comparing structure-function relationships based on Drasdo's and Sjostrand's retinal ganglion cell displacement models. *Invest Ophthalmol Vis Sci*. 2020; 61: 10. 10.1167/iovs.61.4.10 PMC7401427 32293667

[24] Atchison DA. Optical models for human myopic eyes. *Vis Res*. 2006; 46: 2236–2250. 16494919 10.1016/j.visres.2006.01.004

[25] Atchison DA, Pritchard N, Schmid KL, Scott DH, Jones CE, Pope JM Shape of the retinal surface in emmetropia and myopia. *Invest Ophthalmol Vis Sci*. 2005; 46: 2698–2707. 16043841 10.1167/iovs.04-1506

[26] Pope JM, Verkicharla PK, Sepehrband F, Suheimat M, Schmid KL, Atchison DA Three-dimensional MRI study of the relationship between eye dimensions, retinal shape and myopia. *Biomed Opt Express*. 2017; 8: 2386–2395. 28663880 10.1364/BOE.8.002386 PMC5480487

[27] Coletta NJ, Watson T. Effect of myopia on visual acuity measured with laser interference fringes. *Vis Res*. 2006; 46: 636–651. 16045959 10.1016/j.visres.2005.05.025

[28] Chui TY, Song H, Burns SA Individual variations in human cone photoreceptor packing density: variations with refractive error. *Invest Ophthalmol Vis Sci*. 2008; 49: 4679–4687. 18552378 10.1167/iovs.08-2135 PMC2710765

[29] Dabir S, Mangalesh S, Schouten JS, et al. Axial length and cone density as assessed with adaptive optics in myopia. *Ind J Ophthalmol*. 2015; 63: 423–426. 10.4103/0301-4738.159876 PMC4501139 26139804

[30] Stapley V, Anderson RS, Saunders KJ, Mulholland P Altered spatial summation optimizes visual function in axial myopia. *Sci Rep*. 2020; 10: 12179. 32699286 10.1038/s41598-020-67893-8 PMC7376210

[31] Chang WC, Joe, Allaire JJ, Xie Y, McPherson J Shiny: Web Application Framework for R. 2019.

[32] RCT. *R: A Language and Environment for Statistical Computing*. Vienna, Austria: R Foundation for Statistical Computing; 2019.

[33] Curcio CA, Sloan KR, Meyers D Computer methods for sampling, reconstruction, display and analysis of retinal whole mounts. *Vis Res*. 1989; 29: 529–540. 2603390 10.1016/0042-6989(89)90039-4

[34] Strasburger H, Rentschler I, Juttner M Peripheral vision and pattern recognition: a review. *J Vis*. 2011; 11: 13. 10.1167/11.5.13 PMC11073400 22207654

[35] Garg A, Hood DC, Pensec N, Liebmann JM, Blumberg DM Macular damage, as determined by structure-function staging, is associated with worse vision-related quality of life in early glaucoma. *Am J Ophthalmol*. 2018; 194: 88–94. 30053467 10.1016/j.ajo.2018.07.011

[36] Hood DC, Tsamis E, Bommakanti NK, et al. Structure-function agreement is better than commonly thought in eyes with early glaucoma. *Invest Ophthalmol Vis Sci*. 2019; 60: 4241–4248. 31618760 10.1167/iovs.19-27920 PMC6860999

[37] Sato S, Hirooka K, Baba T, Tenkumo K, Nitta E, Shiraga F Correlation between the ganglion cell-inner plexiform layer thickness measured with cirrus HD-OCT and macular visual field sensitivity measured with microperimetry. *Invest Ophthalmol Vis Sci*. 2013; 54: 3046–3051. 23580483 10.1167/iovs.12-11173

[38] Ohkubo S, Higashide T, Udagawa S, et al. Focal relationship between structure and function within the central 10 degrees in glaucoma. *Invest Ophthalmol Vis Sci*. 2014; 55: 5269–5277. 25082882 10.1167/iovs.14-14153

[39] Sample PA, Dannheim F, Artes PH, et al. Imaging and Perimetry Society standards and guidelines. *Optom Vis Sci*. 2011; 88: 4–7. 21099442 10.1097/OPX.0b013e3181fc3735

